# Effects of open-label placebos and self-monitoring in skin-picking disorder: a randomized crossover trial

**DOI:** 10.3389/fpsyt.2025.1645958

**Published:** 2025-09-04

**Authors:** Anne Schienle, Albert Wabnegger, Sandra Tanzmeister

**Affiliations:** Clinical Psychology, University of Graz, Graz, Austria

**Keywords:** open-label placebo, skin-picking, app-assisted, self-monitoring, body-focused repetitive behaviors (BFRBs)

## Abstract

**Background:**

Symptom reduction in skin-picking disorder (SPD) has previously been demonstrated using deceptive placebo treatments. However, to date, no study has investigated the effects of open-label placebos (OLPs) in this context.

**Method:**

Fifty-two participants (mean age = 30 years, 85% female) with pathological skin-picking took part in a clinical crossover trial. The study included daily app-assisted symptom monitoring over two conditions: two weeks of OLP treatment (one pill per day) and two weeks without OLP treatment. No pharmacological treatment was given to reduce skin-picking during the trial. Symptom severity was measured using a disorder-specific questionnaire administered at baseline and after each two-week condition (OLP, no OLP) along with daily app ratings of symptom severity.

**Results:**

Both self-monitoring with and without OLP treatment resulted in reduced questionnaire scores compared to baseline. Daily app-based ratings showed a modest reduction (-3%) in the urge to engage in skin-picking and the time spent picking (-6 minutes/day) during the OLP condition. Participants generally adhered well to the pill-taking protocol, though many were skeptic toward the OLP treatment.

**Conclusion:**

When used as a stand-alone-intervention, OLP treatment demonstrated only minimal effects beyond those achieved through self-monitoring.

## 1 Introduction

Skin-picking disorder (SPD) is classified under the category of obsessive-compulsive and related disorders in the DSM-5 (1). Key diagnostic criteria include recurrent and excessive skin manipulation (e.g., scratching, rubbing, squeezing) resulting in physical complications (e.g., wounds, infections, scarring), psychological distress, and functional impairment. SPD is a common mental health condition, affecting approximately 2–5% of the general population, with a higher prevalence observed in females compared to males (1, 2).

Both pharmacological and nonpharmacological approaches are available for the treatment of SPD (for systematic reviews and meta-analyses see 3–6). Among the medications studied, selective serotonin reuptake inhibitors (SSRIs) have shown the most promising results in reducing skin-picking symptoms (6).

Nonpharmacological interventions for SPD focus on psychoeducation, habit reversal training, cognitive restructuring, and relapse prevention through enhancement of self-efficacy – often combined in cognitive behavioral therapy (4, 7). Moreover, therapies that incorporate techniques such as mindfulness and emotion regulation training have produced symptom reduction (e.g., 3, 8). Their effectiveness is unsurprising, given that individuals with excessive skin-picking often struggle with emotion regulation and engage in skin manipulation as a means of alleviating negative affect. This mechanism has been described in the emotion regulation theory of SPD by Snorrason et al. (9), the most widely accepted explanation for this disorder.

However, existing treatments for SPD are not universally accessible, may be time-consuming or costly, and can have adverse side effects. Therefore, there is an urgent need for alternative or complementary interventions. One promising approach to addressing a core mechanism of SPD – emotion regulation deficits – is placebo treatment. Placebos, such as inert pills presented as anxiolytics or antidepressants, have been shown to reduce emotional distress in patients with depression and anxiety disorders (10). They have been used both as partial substitutes for active medications (dose extension) and to enhance the effects of psychotherapy (11).

Beyond anxiety and depression, placebo treatments have also shown efficacy in alleviating symptoms of several other mental disorders. Notably, a recent study reported significant effects of placebo treatment on pathological skin-picking (12). In this randomized crossover trial, participants with a primary diagnosis of SPD (n = 69; 90% female; M = 31 years) experienced a clinically meaningful reduction in skin-picking symptoms after two weeks of daily placebo administration. The placebo was deceptively presented as N-acetylcysteine (NAC), a glutamate modulator known for its therapeutic potential in SPD (6, 13, 14). In addition to symptomatic improvement, participants also reported decreased perceived stress and fewer difficulties with emotion regulation following the placebo intervention.

Since deceptive placebo treatment cannot be integrated into everyday clinical practice due to ethical concerns, another placebo approach was used for the present investigation: Open-label placebo (OLP) treatment. OLPs are administered with full transparency; patients are explicitly informed that they are receiving an inert substance. This treatment is typically introduced with a rationale explaining why OLPs can be effective, along with positive verbal suggestions aimed at promoting symptom improvement (15). The mechanisms through which OLPs exert their effects are still under investigation. Proposed explanations include the retrieval of pharmacological memory, (subconscious) conditioning, conscious expectancy, and embodiment. The concept of embodiment refers to the beneficial effects derived from the physical act of taking a pill—a learned, meaningful ritual that is associated with healing (15).

Accumulating evidence shows that OLPs are helpful for a variety of psychological and physical conditions (16, 17). In their meta-analysis, von Wernsdorff et al. (16) found moderate effects of OLPs on subjective complaints but note there might be a bias towards the publication of more “positive” studies.

A closer examination of clinical trials involving OLPs reveals several issues that can affect the interpretation of findings. One key concern is the type of OLP prescription. In many studies, OLP treatment is not administered as a stand-alone intervention; rather, it is often combined with other therapeutic approaches, making it difficult to isolate the specific effects of the placebo. For instance, in a highly cited OLP study by Kaptchuk et al. (18) on irritable bowel syndrome (IBS), 54% of patients in the OLP group were also taking IBS medication, and 24% were on antidepressants. Thus, OLPs functioned as complementary treatment.

Moreover, OLP treatment in clinical trials is often accompanied by increased symptom monitoring. OLPs are typically administered at least once daily over an extended period, during which patients are asked to regularly report on their symptoms. This routine directs attention toward the targeted symptoms and their changes over time. Research has shown that self-monitoring alone can lead to a reduction in maladaptive behaviors (e.g., alcohol use, smoking) as well as symptoms of depression and anxiety (e.g., 19–22). It has been proposed that monitoring techniques enhance patients’ emotional self-awareness, thereby improving their capacity for self-regulation (20). However, the potential influence of self-monitoring on outcomes in OLP trials has not yet been systematically investigated.

Therefore, the present randomized cross-over trial investigated the efficacy of a two-week OLP intervention (one pill per day) in patients with SPD in comparison to no OLP treatment for two weeks (control condition). The study included daily app-assisted self-monitoring (evaluation of the urge to engage in skin-picking, the time spent picking, and perceived stress). At baseline (before treatment), and after the two conditions (OLP, no OLP), participants completed a disorder-specific questionnaire and additional scales (perceived stress, difficulties in emotion regulation, psychological distress). Participants did not take any pharmacological interventions for skin-picking during the course of the study. It was hypothesized that OLP treatment (compared to no OLP) would reduce self-reported symptoms of pathological skin-picking (questionnaire scores, app ratings).

## 2 Method

### 2.1 Participants

Participants with pathological skin-picking were recruited through self-help groups for body-focused repetitive behaviors, social media platforms, and the outpatient clinic of the university. The use of OLPs as a potential intervention for reducing SPD symptoms was clearly disclosed in the study invitation.

A total of 131 participants completed the initial questionnaire survey. Twenty-two participants were excluded because they did not meet the inclusion criteria, or met any exclusion criteria. The remaining 109 participants were enrolled in the study. Inclusion criteria were age > 18 years and a score ≥ 7 (clinical cut-off) on the Skin Picking Scale-Revised (SPS-R; 26); exclusion criteria were reported psychotic symptoms, substance abuse, severe depression, and a diagnosis of borderline personality disorder. Fifty-two participants (44 female, 7 male, and 1 non-binary; 92% with a high school diploma) completed the study. The mean age was 29.69 years (SD = 10.6). A CONSORT diagram is provided in **Supplementary Figure S1**.

### 2.2 Questionnaires

a. The German version of the Skin Picking Scale-Revised (SPS-R; 26) comprises eight items (e.g., “How strong was your urge to pick at your skin?”) that are answered on a 5-point Likert scale (0 = “no urge”; 4 = “very strong urge”). Higher scores indicate greater symptom severity (Cronbach’s alpha: baseline = .80).
b. The Difficulties in Emotion Regulation Scale (DERS; 23) is a 36-item instrument that is divided into six subscales. In the present study, three subscales (Difficulty Engaging in Goal-Directed Behavior, Impulse Control Difficulties and Limited Access to Emotion Regulation Strategies) were utilized (based on a previous study with deceptive placebos). The items are rated on a 5-point Likert scale (1 = “almost never”, 5 = “almost always”). A mean score was computed. Higher scores indicate greater difficulties with emotion regulation (Cronbach’s alpha: baseline = .82).
c. The Brief Symptom Inventory (BSI-18; 24) assesses symptoms of somatization, depression, and anxiety with a total of 19 items rated on a 5-point Likert scale (0 = “not at all” to 4 = “very strongly”). A total score indexing psychological distress was computed (Cronbach’s alpha: baseline = .88).
d. The short version of the Perceived Stress Questionnaire (PSQ-20; 25) consists of 20 items (e.g., “Your problems seemed to be piling up.”) that are rated on a 4-point Likert scale (from 1=“almost never” to 4=“most of the time”). Higher scores indicate greater stress (Cronbach’s alpha, baseline= .74).

In addition to the questionnaires, the expected effect of the OLP (before treatment) and the perceived effect of the OLP (end of study) were rated on scales ranging from 0 (“not effective”) to 100 (“very effective”).

### 2.3 Design and procedure

**Figure 1:**
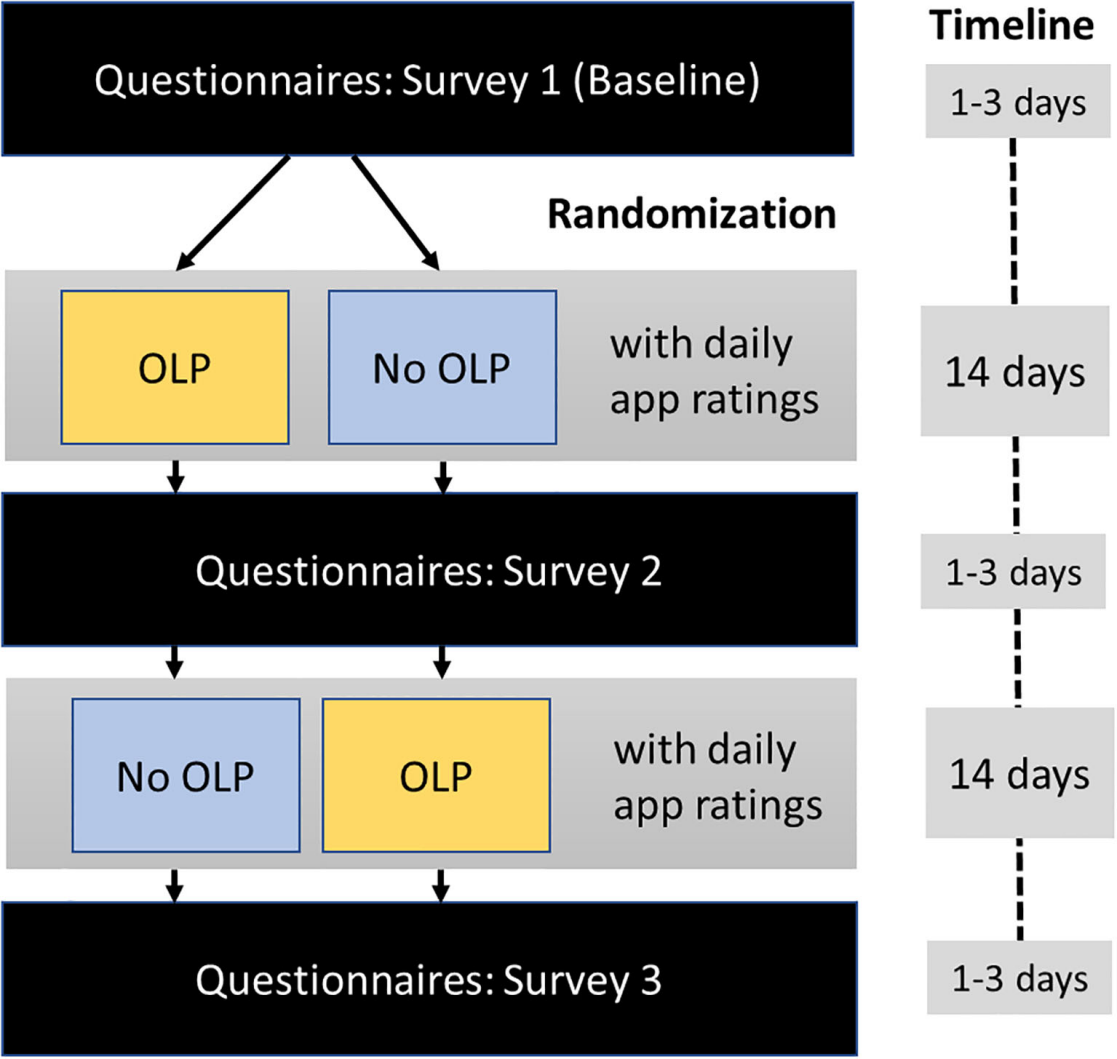
Study procedure and timeline. Surveys included the skin-picking scale-revised, the difficulties in emotion regulation scale, the brief symptom inventory, and the perceived stress questionnaire; daily app ratings included urge to engage in skin-picking, time spent picking, stress level, and pill taking (OLP period only).

The study was conducted between October, 1^st^, 2024 and February, 9^th^, 2025 and organized through the outpatient clinic of the University of Graz (Austria) which has a specialized unit for SPD. Participants first answered the questionnaires (baseline survey; **Figure 1**). Then, they were randomly assigned to one of two sequences of experimental conditions: OLP – No OLP or No OLP – OLP (random number table).

Before the OLP condition, participants received written information about the concept of OLPs and positive results from scientific OLP studies (**Supplementary Material S1**). A container (size 35 x 70 mm, 50 ml) filled with 14 placebo capsules (size 00; filled with starch) for daily intake in the morning was sent via mail to each participant. The container had the label “Placebo – for reducing skin-picking”.

Each condition (OLP, No OLP) lasted two weeks, where participants used a smartphone app (Vue, a progressive web application) to complete daily self-reports. Each evening they documented their urge to engage in skin-picking (“How strong was your urge to pick at your skin today? (0: “not present”; 100: “very strong”), their stress level (“How stressed were you today?” (0: “not at all”; 100: “very much”), the amount of time spent picking each day (in hours), and whether they had taken the pill (OLP condition only). Moreover, they could leave open comments. At the end of each two-week period, participants completed the set of questionnaires (survey) again (**Figure 1**).

During the trial, all communication with participants was conducted via email. Approximately 1–3 days after completing the baseline survey, participants began their first assigned condition (OLP or No OLP) and received condition-specific instructions. After seven days, they were sent a reminder email to continue completing the app-based ratings. At the end of the 14-day period, participants received a link to the second questionnaire survey. Upon completing this survey, participants received instructions for the second condition (OLP or No OLP, depending on their initial assignment). As with the first phase, they received a reminder email after seven days and a link to the final (third) survey after 14 days. If a survey was not completed, participants received an email reminder after two days.

The app did not include any prompts, notifications, or feedback when one or more entries were missed. We chose this approach because we were also interested in examining the effects of the OLP on participants’ compliance with app usage.

This OLP study followed the Declaration of Helsinki and was approved by the local ethics committee (GZ. 39/223/63 ex 2023/24). The trial was preregistered at the German Clinical Trials Register (DRKS00035003).

### 2.4 Statistical analysis

#### 2.4.1 Questionnaire data

Repeated measures one-way analysis of variance (ANOVA) compared the questionnaire scores (SPS_R, DERS, PSQ, BSI) between the three timepoints (surveys at baseline, post OLP, post No OLP). The scores of the two sequence groups were combined separately for the OLP and No OLP conditions. Significant effects were followed up by paired t-tests (Bonferroni correction 0.05/3 = 0.016). Effect sizes were calculated using η2p and Cohen’s d. All 52 participants completed the surveys at each of the three timepoints.

#### 2.4.2 App ratings

App ratings (urge to engage in skin picking, time spent picking, perceived stress) were analyzed using linear mixed models with random intercepts for participants (i.e., DV ~ 1 + condition + (1 I id)). Including random slopes did not improve model fit and were therefore omitted. Results were considered as statistically significant when p <.05.

Analyses were conducted with JAMOVI (2.6.17), including the GAMLj-package and IBM SPSS Statistics version: 29.0.0.0 (241). The study initially aimed to test 100 participants (see preregistration); 52 participants completed the study. A *post-hoc* sensitivity analysis using G*Power (version 3.1) for a repeated-measures ANOVA with three time points (α = .05, power = .80, n = 52) indicated that the study was powered to detect a minimum effect size of f = 0.18, corresponding to a small-to-medium effect.

## 3 Results

### 3.1 Questionnaire data

#### 3.1.1 SPS_R

**Figure 2:**
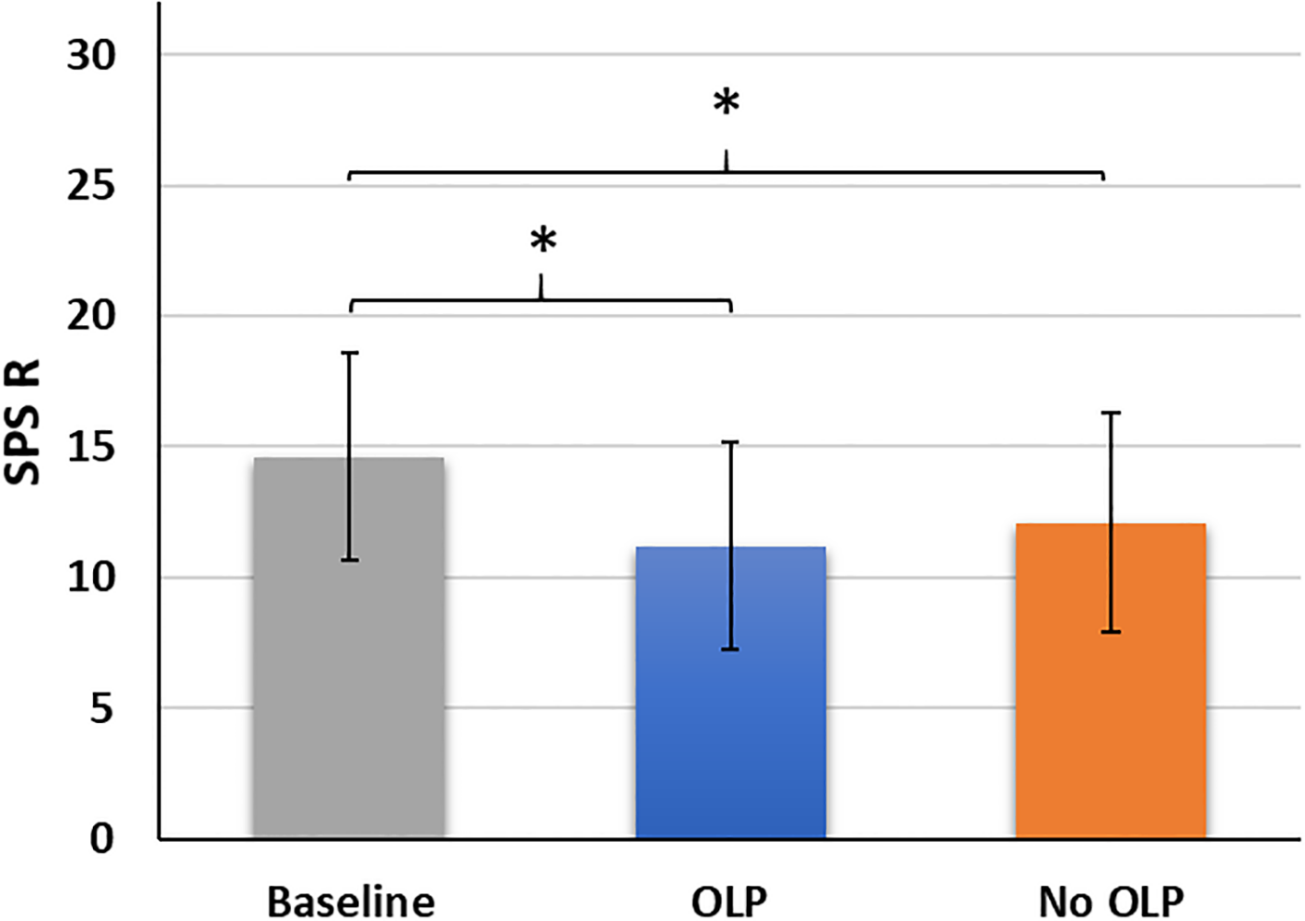
Mean scores (standard deviations) of the skin-picking scale revised (SPS_R) across the three timepoints baseline, after two weeks of open-label placebo (OLP) treatment, after two weeks with no OLP treatment. *p <.05..

Mean scores differed significantly across the three survey timepoints (F(2,102) = 29.15, p <.001, partial η² = .36). Baseline scores (before treatment) were higher than those after two weeks of OLP treatment (t(51) = 7.95, p <.001, Cohen’s d = 1.10) and after two weeks without OLP treatment (t(51) = 5.21, p <.001, Cohen’s d = 0.72). No difference was observed between OLP and No OLP (t(51) = 1.86. p = .068, Cohen’s d = 0.26; **Figure 2**).

#### 3.1.2 DERS

Mean scores did not differ across the three survey timepoints: before treatment (baseline), after OLP treatment and after No OLP treatment (F(2,102) = 2.30, p = .105, partial η² = .043).

#### 3.1.3 PSQ

Mean scores did not differ across the three survey timepoints (F(2,102) = 0.99, p = .375, partial η² = .019).

#### 3.1.4 BSI

Mean scores significantly differed across the three survey timepoints (F(2,102) = 4.80, p = .009, partial η² = .09). Scores after two weeks of OLP treatment were lower than those at Baseline (t(51) = 3.04, p = .004, d =0.42). No significant differences were observed between OLP and No OLP (t(51) = 1.83, p = .073, d = 0.25), and the No OLP and Baseline (t(51) = 1.17, p = .249, d = 0.16). For descriptive statistics see **Supplementary Table S1**.

The two sequence groups (OLP – No OLP *vs*. No OLP – OLP) did not significantly differ in their baseline scores for neither of the questionnaires (all p’s ≥.08; see **Supplementary Table S2** for t-statistics of baseline comparisons as well as means and standard deviations across all time points for the two sequence groups).

### 3.2 App ratings

#### 3.2.1 Urge to engage in skin picking

The linear mixed model revealed a statistically significant main effect of condition (F(1,1077.23) = 7.64, p = .006) indicating a slightly reduced urge to engage in skin picking (M = 33.47, SE = 2.04) during the OLP relative to the no OLP condition (M = 36.74, SE = 2.05; see **Supplementary Figure S1**).

#### 3.2.2 Reported time for engaging in skin-picking

The main effect for condition was statistically significant (F(1,1073.25) = 4.04, p = .045). Reported time for skin-picking per day (in hours) was lower in the OLP condition (M = 1.08, SE = 0.05) than in the no OLP condition (M = 1.18, SE = 0.05; see **Supplementary Figure S2**).

#### 3.2.3 Stress level

The main effect of condition (OLP, No OLP) did not reach statistical significance (F(1, 1077.15) = 0.02, p = .887).

#### 3.2.4 Open comments

No adverse side effects were reported by the participants.

### 3.3 Perceived treatment efficacy

Expected efficacy of the OLP before treatment (M = 31.38, SD = 21.03) did not differ from the perceived efficacy at the end of the trial (M = 27.96, SD = 25.76, t(51) = 0.91, p = .37, d = 0.13). Expected efficacy was not correlated with the net OLP effect (SPS-R: No OLP condition minus OLP condition r = -.001, p = .994; app ratings: urge to pick in No OLP condition minus OLP condition; r = .03, p = .815).

### 3.4 Treatment adherence

The number of completed app ratings did not differ between the OLP condition (M = 10.94, SD = 2.52) and No OLP condition (M = 10.65, SD = 3.53; t(51) = .77, p = .45, d = 0.11). The number of pills taken had a median of 11 (IQR 8 – 12) and a mean of M = 10.33 (SD = 2.68); 39% of participants showed high adherence, reporting that they had taken at least 12 pills. Only 6% of participants reported low adherence (only 5 or less pills taken).

We also conducted a dropout analysis. Baseline questionnaire scores (survey 1) were compared between those participants who completed the study (n = 52) and those who did not (n = 57). The two groups did neither differ in any of the assessed questionnaires (**Supplementary Table S3**) nor in mean age (t(107) = - 1.17, p = 0.245).

## 4 Discussion

This crossover clinical trial with daily symptom monitoring examined the effectiveness of open-label placebo (OLP) treatment in reducing pathological skin-picking. Participants completed a symptom-specific questionnaire (SPS_R, 26) at baseline, after a two-week period of daily OLP pill treatment, and after a two-week control period without OLP administration. Results indicated that both the OLP and No OLP condition led to significant reductions in SPS_R scores compared to baseline. There was no significant difference in reported symptom severity between OLP and No OLP. The analysis of app-based daily ratings revealed that participants’ urge to engage in skin-picking was only slightly lower (minus 3%) on days when the OLP pill was taken, and the reported time spent picking decreased by about six minutes per day. Additional questionnaires and ratings concerning perceived stress or difficulties with emotion regulation indicated no OLP effect.

These findings are unexpected in light of previous clinical OLP trials, which have demonstrated small to moderate effects on patients’ self-reported symptoms (see meta-analyses by 16 and 17). However, the current study differs from earlier research in several key aspects. Most prior trials administered OLP treatment in addition to treatment as usual (TAU) and compared this combination to TAU alone (e.g., 27–29). In these cases, OLPs functioned as adjunctive treatments rather than stand-alone intervention, as was the case in the present study. Evaluating OLPs as the sole treatment allows for an assessment of their net effect – an effect that, in this study, proved to be negligible.

The present study incorporated ecological momentary assessment (EMA), allowing participants to evaluate their symptoms on a daily basis. Findings indicated that this self-monitoring alone had a beneficial effect in reducing pathological skin-picking. Symptom tracking – along with associated processes such as emotions, cognitions, and overt behaviors is a well-established method in clinical practice. This type of behavioral analysis belongs to standard diagnostics in Cognitive Behavioral Therapy (CBT) and aims at mapping out and understanding the problematic behaviors of the patient (30). It can enhance patients’ awareness of previously unnoticed patterns, particularly regarding how their dysfunctional behaviors (i.e., skin-picking) is triggered and maintained. The increased self-directed awareness may also interrupt automatic behaviors, such as unconscious skin-picking. The observed positive effects of self-monitoring in the current study are in line with the self-awareness theory (31), which states that when people focus attention on themselves, they compare their current behavior to internal standards or goals. Engaging in self-monitoring increases their awareness of discrepancies, prompting corrective actions. The present findings also align with empirical evidence as obtained by an internet-based self-help program for SPD that included multiple treatment components (32). During the first two weeks of the program, participants were instructed to keep a “picking diary” to establish a baseline. The self-monitoring alone already led to a reduction in skin-picking symptomatology.

Moreover, this trial examined the effects of remotely administered OLPs, a topic that has been rarely explored. In a study by Guevarra et al. (33), remotely administered OLP treatment significantly reduced COVID-related stress, anxiety, and depression, suggesting that close personal interaction may not be essential to achieve symptom improvement. Similar to the present investigation, the authors mailed OLP pills to participants for daily use over a two-week period. However, Kube et al. (34) found that when participant contact was limited – as opposed to enhanced – OLP effects on allergic rhinitis were diminished.

It has to be noted that adherence to the OLP protocol was good in the present investigation. On average, participants took 10 out of 14 pills (according to the daily app ratings). This is partly in line with previous reports. For example, an OLP study on premenstrual syndrome (35) also reported good adherence to the OLP protocol. Moreover, the authors reported high acceptability of the OLP approach. This was different in the present study. On average, participants expressed modest expectations concerning the treatment, and also perceived only small effects of the OLP intervention. Positive expectations are, however, considered an important mechanism through which OLPs exert their effects (15). Low perceived effectiveness and even disappointment with OLP treatment has been reported before in clinical trials (e.g., in patients diagnosed with depression; 29). For future studies, it would be important to enhance positive expectations regarding OLPs (e.g. via extended psychoeducation), or to select patients based on their expectations before the treatment.

We also have to mention the following limitations of the present study. First, as SPD is more common in women, our sample was predominantly female, therefore the findings cannot be generalized to the male population. Second, reported symptoms were only moderate concerning experienced urge to manipulate one’s skin; OLP effects might be more pronounced in individuals with higher symptom severity (e.g., 17). Third, the scale used to assess time spent on skin-picking (in hours) was not optimal for detecting placebo-induced changes, as it lacked sufficient granularity. Fourth, half of participants who enrolled in the study (and started with the first questionnaire assessment) did not finish the trial (despite email reminders). A similar dropout rate (47%) was observed in a deceptive placebo (DP) study on reducing pathological skin-picking (12), which used the same design and procedure as this OLP study. However, in contrast to the OLP trial, participants in the DP study reported substantial symptom improvement.

The current clinical trial also has two main strengths, including the investigation of OLP effects in the absence of other current medication for pathological skin-picking and a repeated-measures design that allowed for control of inter-individual differences.

## 5 Conclusion

When used as a stand-alone-intervention, OLP treatment demonstrated only minimal effects beyond those achieved through self-monitoring. Future studies should further elucidate whether OLPs may have specific indications limited to dose extension and adjunctive therapy.

## Data Availability

The original contributions presented in the study are included in the article/**Supplementary Material**. Further inquiries can be directed to the corresponding author.
