# Supplementary material for: Effects of open-label placebos and self-monitoring in skin-picking disorder: a randomized crossover trial
Source: Front Psychiatry. 2025 Sep 4;16:1645958. doi: 10.3389/fpsyt.2025.1645958 (PMC12443836; doi:10.3389/fpsyt.2025.1645958)

**Supplementary Figure S1:** CONSORT diagram

**
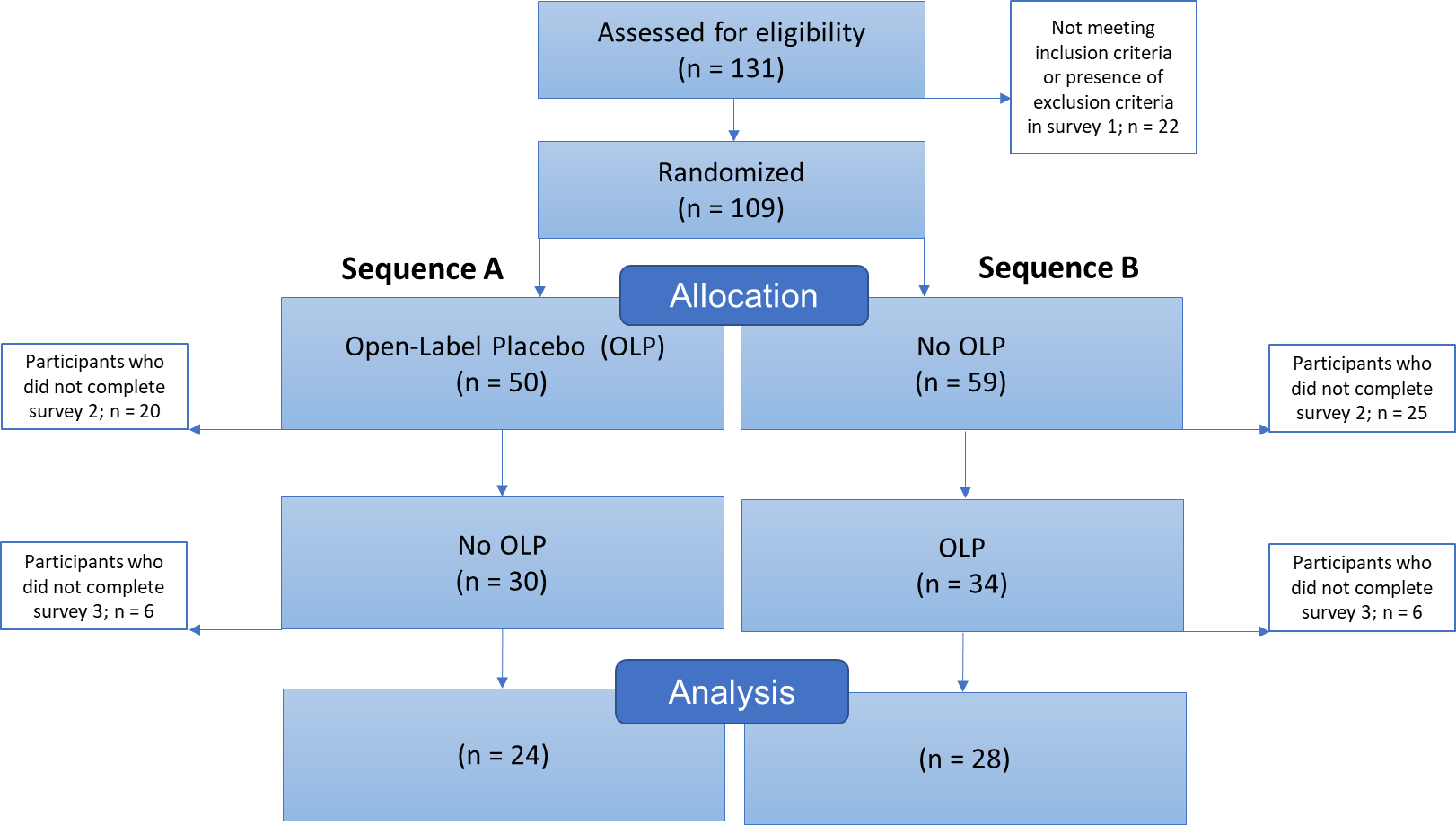
**

**Supplementary Material S1:** Packaging leaflet (translation)

Dear Participant,

Thank you for taking part in our study on the effectiveness of open-label placebos (OLPs) for dermatillomania (skin-picking disorder).

In this information leaflet, you will find details about OLPs, usage instructions and guidance as well as a link to install the study app.

INFORMATION ABOUT THE OPEN PLACEBO:

A placebo pill has no specific effect on the condition or symptoms being treated. Typically, placebos are administered “covertly,” meaning the recipient is led to believe it is an active medication. In the case of an “open-label placebo” (OLP), however, the patient is fully aware that they are taking a placebo.

Research findings¹ have shown that placebo effects can occur not only when patients mistakenly believe they are receiving medication, but also when they are informed beforehand that it is a placebo. For example, physician Ted J. Kaptchuk (Boston, USA) conducted a study involving open-label placebos with 80 patients diagnosed with irritable bowel syndrome². The patients in the placebo group experienced significantly greater relief from their symptoms compared to those in the control group (who had the same quality of clinical contact but no placebo). This unconventional therapeutic approach was explained as follows: clinical research has demonstrated that placebos can support mind-body self-healing processes.

The underlying mechanisms of open-label placebos are not yet fully understood. However, they are likely very similar to those of traditional covert placebos: positive expectations and prior learned experiences (with medications) can initiate beneficial changes in thoughts, emotions, and physical processes.

An international panel of experts on the clinical use of placebos³ recommends prescribing open-label placebos over deceptive placebos when there is evidence supporting the effectiveness of placebos for a particular condition. As our research has shown, this is the case for skin-picking disorder.

REFERENCES:

von Wernsdorff, M., Loef, M., Tuschen-Caffier, B., & Schmidt, S. (2021). Effects of open-label placebos in clinical trials: a systematic review and meta-analysis. Scientific Reports, 11(1), 3855.

Kaptchuk, T. J., Friedlander, E., Kelley, J. M., Sanchez, M. N., Kokkotou, E., Singer, J. P., ... & Lembo, A. J. (2010). Placebos without deception: a randomized controlled trial in irritable bowel syndrome. PLoS One, 5(12), e15591.

Evers, A. W., Colloca, L., Blease, C., Annoni, M., Atlas, L. Y., Benedetti, F., ... & Kelley, J. M. (2018). Implications of placebo and nocebo effects for clinical practice: expert consensus. Psychotherapy and Psychosomatics, 87(4), 204–210.

COMPOSITION:

One vegan capsule contains: 600 mg potato starch

Excipients: Hydroxypropylmethylcellulose (HPMC)

Free from: GMOs, soy, yeast, gluten, lactose, added sucrose, gelatin, animal products, preservatives, artificial colorings, fragrances, and flavorings.

INSTRUCTIONS FOR USE:

Take one capsule daily in the morning for 14 days.

APP LINK AND INSTALLATION INSTRUCTIONS:

URL: https://olp.datmotion.com/

You can install the link on your smartphone as an app as follows:

Android:

Open the link, click on the three-dot menu in your browser, and select “Add to Home screen.”

iOS:

Open the link in your browser, tap the “Share” icon (bottom center), and select “Add to Home Screen.”

USAGE INSTRUCTIONS:

You can only complete the app once per day. Please do this in the evening or near the end of your day. Also, ensure that you always complete the app on the same device (phone or tablet).

**Supplementary Table S1:** Means (standard deviations) and [95 % Confidence Intervals] for questionnaire scores before treatment (baseline), after the 2-week OLP treatment and after the 2 weeks with no OLP treatment; n = 52

|  | **Baseline** | **OLP** | **No OLP** |
| --- | --- | --- | --- |
| SPS_R | 14.62 (3.95)  [13.52 – 15.71] | 11.21 (3.95)  [10.11 – 12.31] | 12.10 (4.22)  [10.92 – 13.27] |
| DERS | 46.40 (13.21)  [42.73 – 50.08] | 43.56 (12.15)  [40.18 – 46.94] | 45.23 (13.53)  [41.46 – 49.00] |
| PSQ | 53.17 (15.68)  [48.81 – 57.54] | 50.87 (17.93)  [45.87 – 55.86] | 53.30 (16.28)  [48.77 – 57.83] |
| BSI (total) | 16.29 (9.60)  [13.62 – 18.96] | 13.85 (7.62)  [11.72 – 15.97] | 15.15 (8.58)  [12.77 – 17.54] |

Note: SPS_R: Skin-Picking Scale revised, DERS: Difficulties in Emotion Regulation Scale, BSI: Brief Symptom Inventory, and PSQ: Perceived Stress Questionnaire

**Table S2:** Mean questionnaire scores (standard deviations) across timepoints separately for the two sequence groups

|  | **OLP – No OLP**  (n = 24) | **No OLP- OLP** (n = 28) | **t-statistics** |
| --- | --- | --- | --- |
|  | M (SD) | M (SD) |  |
| **SPS_R_T1** | 14.63 (3.98) | 14.61 (3.99) | t(50) = .02, p = .987, d = .004 |
| **SPS_R_T2** | 11.71 (3.95) | 13.00 (4.31) |  |
| **SPS_R_T3** | 11.04 (3.94) | 10.79 (3.97) |  |
|  |  |  |  |
| **DERS_T1** | 48.79 (14.31) | 44.36 (12.08) | t(50) = 1.21, p = .231, d = .337 |
| **DERS_T2** | 47.04 (12.75) | 41.04 (12.40) |  |
| **DERS_T3** | 50.13 (13.38) | 40.57 (10.97) |  |
|  |  |  |  |
| **PSQ_T1** | 57.29 (15.56) | 49.64 (15.18) | t(50) = 1.79, p = .079, d = .498 |
| **PSQ_T2** | 56.11 (14.70) | 49.58 (15.47) |  |
| **PSQ_T3** | 57.63 (16.44) | 46.37 (19.44) |  |
|  |  |  |  |
| **BSI_T1** | 17.75 (10.05) | 15.04 (9.20) | t(50) = 1.02, p = .314, d = .283 |
| **BSI_T2** | 14.50 (6.20) | 14.14 (9.19) |  |
| **BSI_T3** | 15.29 (7.99) | 13.29 (8.74) |  |

Note: SPS_R: Skin-Picking Scale revised, DERS: Difficulties in Emotion Regulation Scale, BSI: Brief Symptom Inventory, and PSQ: Perceived Stress Questionnaire; T1: baseline; T2: after OLP or No OLP treatment; T3: after OLP or No OLP treatment – depending on sequence

**Table S3:** Comparison of mean questionnaire scores (standard deviations) between enrolled participants who completed the study vs. dropped out

|  | **Completers (N = 52) M (SD)** | **Dropouts (N = 57) M (SD)** | **t-statistics** |
| --- | --- | --- | --- |
| SPS_R | 14.62 (3.95) | 15.05 (5.14) | *t*(107) = 0.494, p = .622 |
| DERS | 46.40 (13.21) | 46.39 (15.64) | *t*(107) = -0.006, p = .995 |
| PSQ | 53.17 (15.68) | 54.33 (19.98) | *t*(107) = 0.333, p = .740 |
| BSI | 16.29 (9.60) | 17.84 (9.20) | *t*(107) = 0.863, p = .390 |

Note: SPS_R: Skin-Picking Scale revised, DERS: Difficulties in Emotion Regulation Scale, BSI: Brief Symptom Inventory, and PSQ: Perceived Stress Questionnaire

**Figure S1**: Means, standard error and random effects for the reduced urge to engage in skin picking (as observed in the app ratings) during OLP relative to the control condition (no OLP)


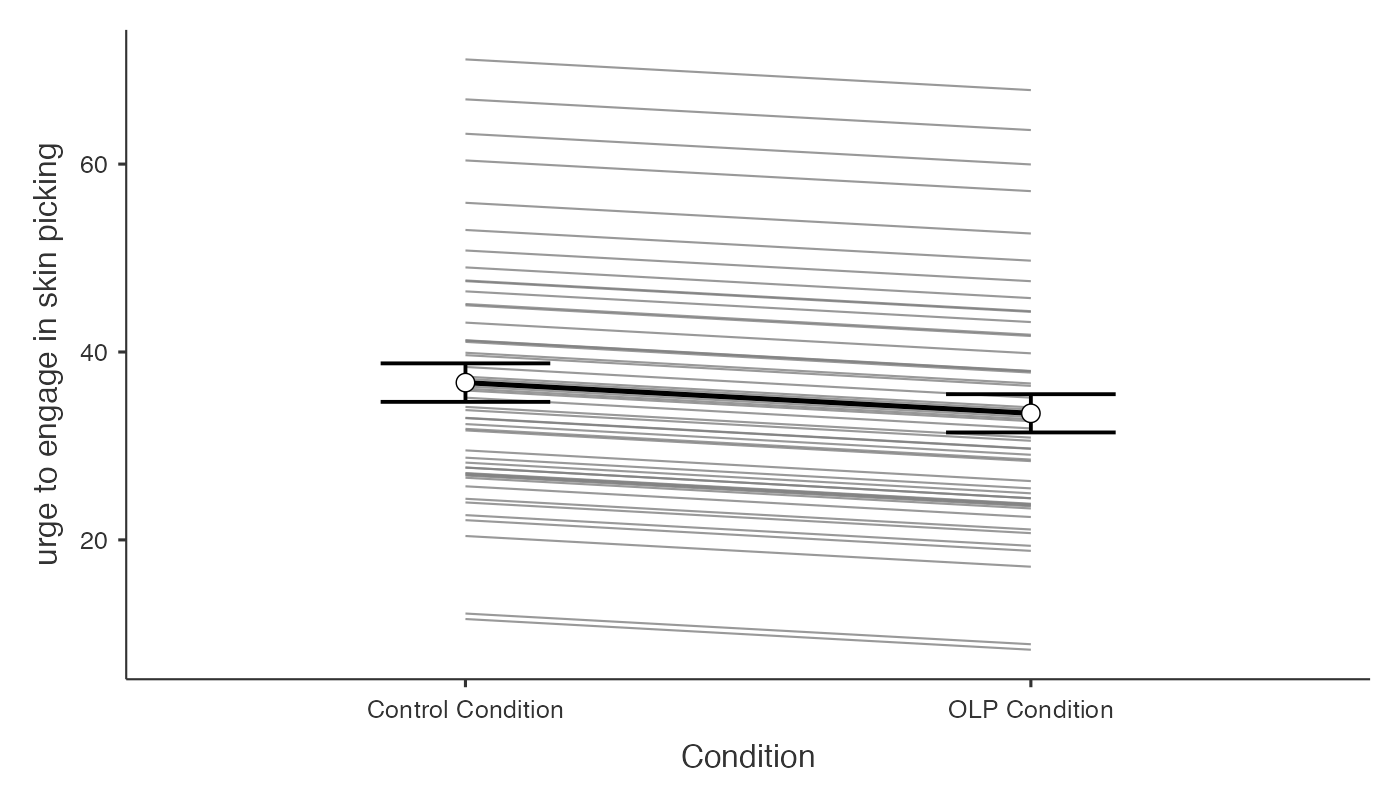


**Figure S2**: Means, standard error and random effects for the reduced time of engaging in skin picking during OLP relative to the control condition (no OLP)


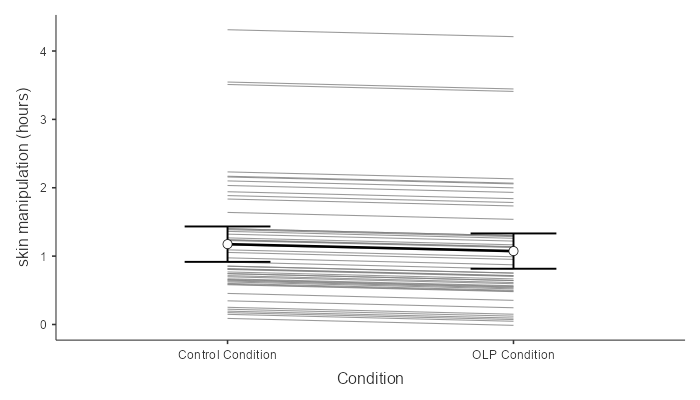

Supplement: Supplementary file 1 [file Supplementaryfile1.docx]
